# Improvement in Uncontrolled Eating Behavior after Laparoscopic Sleeve Gastrectomy Is Associated with Alterations in the Brain–Gut–Microbiome Axis in Obese Women

**DOI:** 10.3390/nu12102924

**Published:** 2020-09-24

**Authors:** Tien S. Dong, Arpana Gupta, Jonathan P. Jacobs, Venu Lagishetty, Elizabeth Gallagher, Ravi R. Bhatt, Priten Vora, Vadim Osadchiy, Jean Stains, Anna Balioukova, Yijun Chen, Erik Dutson, Emeran A. Mayer, Claudia Sanmiguel

**Affiliations:** 1 The Vatche and Tamar Manoukian Division of Digestive Diseases, Department of Medicine, David Geffen School of Medicine at UCLA, Los Angeles, CA 90095, USA; tsdong@mednet.ucla.edu (T.S.D.); agupta@mednet.ucla.edu (A.G.); jjacobs@mednet.ucla.edu (J.P.J.); Vlagishetty@mednet.ucla.edu (V.L.); Emayer@mednet.ucla.edu (E.A.M.); 2 UCLA Microbiome Center, David Geffen School of Medicine at UCLA, Los Angeles, CA 90095, USA; 3 Division of Gastroenterology, Hepatology and Parenteral Nutrition, VA Greater Los Angeles Healthcare System, Los Angeles, CA 90025, USA; 4 G. Oppenheimer Center for Neurobiology of Stress and Resilience, University of California, Los Angeles, CA 90095, USA; lgallagher18@ucla.edu (E.G.); rbhatt@usc.edu (R.R.B.); PPVora@mednet.ucla.edu (P.V.); VOsadchiy@mednet.ucla.edu (V.O.); Jstains@mednet.ucla.edu (J.S.); 5 David Geffen School of Medicine, University of California, Los Angeles, CA 90095, USA; YijunChen@mednet.ucla.edu (Y.C.); EDutson@mednet.ucla.edu (E.D.); 6 Imaging Genetics Center, Mark and Mary Stevens Neuroimaging and Informatics Institute, Keck School of Medicine at USC, University of Southern California, Los Angeles, CA 90033, USA; 7 Department of Urology, David Geffen School of Medicine at UCLA, Los Angeles, CA 90095, USA; 8 UCLA Center for Obesity and METabolic Health (COMET), Los Angeles, CA 90024, USA; abalioukova@mednet.ucla.edu

**Keywords:** bariatric surgery, brain–gut–microbiome axis, metabolite, obesity, brain

## Abstract

Background: Bariatric surgery is proven to change eating behavior and cause sustained weight loss, yet the exact mechanisms underlying these changes are not clearly understood. We explore this in a novel way by examining how bariatric surgery affects the brain–gut–microbiome (BGM) axis. Methods: Patient demographics, serum, stool, eating behavior questionnaires, and brain magnetic resonance imaging (MRI) were collected before and 6 months after laparoscopic sleeve gastrectomy (LSG). Differences in eating behavior and brain morphology and resting-state functional connectivity in core reward regions were correlated with serum metabolite and 16S microbiome data. Results: LSG resulted in significant weight loss and improvement in maladaptive eating behaviors as measured by the Yale Food Addiction Scale (YFAS). Brain imaging showed a significant increase in brain volume of the putamen (*p*.adj < 0.05) and amygdala (*p*.adj < 0.05) after surgery. Resting-state connectivity between the precuneus and the putamen was significantly reduced after LSG (*p*.adj = 0.046). This change was associated with YFAS symptom count. *Bacteroides*, *Ruminococcus*, and *Holdemanella* were associated with reduced connectivity between these areas. Metabolomic profiles showed a positive correlation between this brain connection and a phosphatidylcholine metabolite. Conclusion: Bariatric surgery modulates brain networks that affect eating behavior, potentially through effects on the gut microbiota and its metabolites.

## 1. Introduction

Over the last decade, obesity and obesity-related comorbidities have become the leading causes of morbidity and mortality in the developed world [1]. It is expected that this trend will continue to rise along with the risk of cardiovascular disease, diabetes mellitus, and other obesity-related diseases [1]. To date, dietary and lifestyle modifications remain one of the main treatments for obesity. Unfortunately, a large proportion of patients will continue to have difficulty maintaining a diet long enough to reach significant and sustainable weight loss. While a variety of pharmacological interventions have had minor successes, bariatric surgery remains one of the few proven methods that is able to accomplish significant and sustained weight loss over time [2]. The two most common surgical procedures for bariatric surgery include Roux-en-Y gastric bypass (RYGB) and laparoscopic sleeve gastrectomy (LSG). Both RYGB and LSG have similar effects on weight loss and have been shown to be associated with lower hunger scores and changes in food preference [3,4]. At baseline, resting-state functional brain magnetic resonance imaging (MRI) studies have shown that patients with obesity have alterations in the connections relating to metabolic sensing and reward processing [5,6]. After bariatric surgery, these connections and alterations in brain signaling often normalize [7,8,9]. However, the exact mechanism by which bariatric surgery can alter appetite, eating behaviors and brain signaling is still poorly understood.

One potential area that can connect bariatric surgery to changes in brain signaling and behavior is the brain–gut–microbiome (BGM) axis. The BGM axis is a tightly regulated bidirectional axis whose derailment is a feature of such diseases as irritable bowel syndrome, autism spectrum disorder, and even obesity [10]. Preclinical models have shown that the gut microbiome is involved in brain development, behavior, and neurotransmitter signaling [11,12,13], and that the brain can alter the gut microbiome via changes in mucus production, motility, and immune signaling [14]. In regards to obesity, alterations in the gut microbiome have been associated with changes in eating behavior, development of metabolic syndrome, and alterations in gastrointestinal hormones such as glucagon-like peptide-1 [10,15,16,17]. Changes in eating behavior and gastrointestinal hormones have also been seen in patients who have undergone bariatric surgery. Several studies have shown that bariatric surgery, including LSG, can lead to sustained and significant changes in the gut microbiome, in eating behaviors, and in satiety signaling [18,19,20]. However, how these changes are connected to brain signaling is not entirely clear. In our study, by using an integrated systems biology approach, we applied morphological and functional resting state brain imaging, microbial analysis, and serum metabolomics to investigate the role of bariatric surgery induced changes on the brain–gut–microbiome axis in regulating eating behaviors.

## 2. Materials and Methods

### 2.1. Patient Selection and Study Design

Patients were recruited from the University of California, Los Angeles Bariatric Surgery Program. Only patients undergoing LSG were included to negate any effect of different surgical procedures on brain and microbiome alterations. We further focused our selection to female patients as female patients have a higher prevalence of food addiction than males [21]. Inclusion criteria included adult patients who were considering bariatric surgery and met eligible criteria for surgery. Because brain morphology differs by handedness and advanced age, we limited the study to include right-handed patients age 18 to 55 years old undergoing laparoscopic sleeve gastrectomy (LSG) following the Guidelines for Clinical Application of Laparoscopic Bariatric Surgery by of American Gastrointestinal and Endoscopic Surgeons (SAGES) [22,23]. Exclusion criteria includes a prior history of major gastrointestinal surgery including weight loss surgery, use of medications known to affect hunger, satiety or intestinal motility, any contraindication to undergo MRI, current or past alcohol or drug abuse, pregnancy, current use of insulin or insulin dependent diabetes, inflammatory bowel disease, irritable bowel syndrome, use of probiotics, or use of antibiotics within 1 month of enrollment. Patients were enrolled into the study before undergoing LSG. Subjects underwent a screening visit and 2 study visits occurring at baseline and at 6 months after surgery. Multimodal magnetic resonance brain imaging (MRI), demographic information, height, weight, measures of eating behavior, questionnaire for anxiety and depression, stool samples for 16S ribosomal RNA sequencing, and serum for metabolomics and inflammatory markers were collected at baseline and at 6 months. All subjects gave their informed consent for inclusion before they participated in the study. The study was approved by the Ethics Committee of the UCLA Institutional Review Board (IRB# 13-001552).

### 2.2. Anthropometrics and Body Composition

A certified nutritionist at the UCLA Clinical & Translational Research Center (CTRC) measured height, weight and Body Mass Index (BMI) using techniques and methods described in the National Health and Nutrition Examination Survey (NHANES III) at baseline and at 6 months after surgery. All measurements were taken in duplicate. 

### 2.3. Patient Questionnaire

To assess for anxiety or depressions, patients were asked the Hospital Anxiety and Depression Scale (HADS). A cutoff of 8 or above was considered as abnormal, similar to prior research [24]. To measure eating behaviors, both the Three-Factor Eating Questionnaire R21(TFEQ-R21) and the Yale Food Addiction Scale (YFAS) were used. The TFEQ-R21 is a 21-question survey that measures three categories of eating behavior: cognitive restraint (CR), uncontrolled eating (UE), and emotional eating (EE). A higher score in each subcategory represents higher restraint for eating, more uncontrolled eating, and a predisposition to emotional eating, respectively [25]. The YFAS is a 25-question survey developed to assess food addiction based on the substance dependence criteria, as found in the Diagnostic and Statistical Manual of Mental Disorder, 4th Edition (DSM-4) [26]. It measures withdrawal, dependence on food, tolerance, continued use despite problems, loss of control, time eating, an inability to cut down, and clinical significant impairment. Food addiction was defined as having a YFAS symptom count ≥3 with clinically significant impairment or distress [21]. 

### 2.4. 16S Ribosomal RNA Gene Sequencing and Analysis

Similar to prior published works, DNA was extracted using the ZymoBIOMICS DNA Microprep Kit (Zymo Research, Irvine, CA, USA) per the manufacturer’s protocol. The V4 region of the 16S ribosomal RNA gene was amplified by PCR and underwent 250 × 2 paired-end sequencing on an Illumina HiSeq (Illumina, San Diego, CA, USA) [27,28]. The sequences were processed using the DADA2 pipeline in R which assigns taxonomy using the SILVA 132 database [29]. After pre-processing in R, the data were incorporated into QIIME 2 version 2019.10 [30]. Amplicon sequence variants were filtered if not present in at least 15% of all samples. Sequence depths ranged from 60,710 to 269,258 per sample.

### 2.5. Magnetic Resonance Imaging Acquisition

All neuroimaging was conducted at baseline and at 6-month follow-up on a 3T Siemens PRISMA at the UCLA Ahmanson-Lovelace Brain Mapping Center. T1 images were acquired using the Magnetization Prepared- Rapid Gradient Echo (MP-RAGE) sequence (TR: 2300 ms, TE: 2.98 ms, TI: 900ms, flip angle: 9°, field of view: 240 × 256, slice thickness: 1 mm, voxel resolution: 1 × 1 × 1 mm) to assess brain structure. A 10-min resting-state fMRI scan was acquired to assess resting-state functional connectivity (TR: 2000 ms, TE: 28 ms, flip angle: 77°, acquisition matrix: 64 × 64, slice thickness: 4mm, voxel resolution: 3.44 × 3.44 × 4 mm, 300 volumes).

### 2.6. Brain Regions of Interest

Regions of interest (ROIs) for the morphometry analysis included the bilateral hypothalamus, nucleus accumbens, amygdala, brainstem, putamen and anterior insula based on past literature [31,32,33,34,35]. The same ROIs were used as seeds for the resting-state functional connectivity analysis to the rest of the brain. 

### 2.7. Magnetic Resonance Imaging Processing: Voxel-Based Morphometry

To determine differences in gray matter from baseline to 6 months, a voxel-based morphometry analysis was completed using FMRIB Software Library (FSL)-voxel-based morphometry (VBM), part of the FSL software package [36]. Brain extraction was first conducted using the robust version of the Brain Extraction Tool (BET), which calls the command multiple times, moving the center of gravity to the true center. All images were then checked manually to confirm the brain had been extracted adequately. All brain images were then segmented into gray matter (GM), white matter (WM) and cerebrospinal fluid (CSF) using FMRIB’s Automated Segmentation Tool (FAST) in FSL [37]. A study specific GM template was then created using affine non-linear registration to the ICBM-152 template. Finally, all GM images were non-linearly registered—including a Jacobian modulation to account for differences in transformation—to the study-specific template [38,39] and smoothed with a 4 mm Gaussian kernel. This created a 4D smoothed image. 

### 2.8. Magnetic Resonance Imaging Processing Resting-State Functional Pair-Wise Connections

Preprocessing for the structural and resting-state functional images were done in SPM12 [40]. The first two volumes were discarded to allow for stabilization of the magnetic field. Slice-timing correction was performed followed by six-degree motion correction alignment. The motion parameters were examined for excessive motion in each degree for motion less than 2 mm. Mean frame-wise displacement (FD) and root mean squared (RMS) realignment estimates were also calculated as robust measures of motion using publicly available MATLAB code from GitHub [41]. Each subject’s T1 images was segmented into gray matter, white matter and cerebrospinal fluid, then normalized into the Montreal Neurological Institute (MNI) space. Resting-state images were then co-registered with their respective anatomical T1 images, resulting in resting-state images normalized in MNI space.

The pre-processed, normalized functional images, along with their normalized T1 images and segmentations were then uploaded to the CONN fMRI toolbox [42]. The Schaefer 400 cortical atlas, Harvard-Oxford Subcortical atlas, and the Ascending Arousal Network brainstem atlas were used as ROI’s in MNI space for each subject. The functional images were then denoised using ordinary least squares regression of potential confounding effects and temporal band-pass filtering (0.008–0.09 Hz). The aCompCor method [43] was used and included noise components from white matter, cerebrospinal fluid, estimated subject-motion parameters, and session effects. Fisher transformed correlations were computed (Z) between the functional time series of all the parcellated regions to derive a 417 × 417 matrix for each participant. The bottom half of the undirected matrix was then concatenated into one vector of each subject representing each ROI pair.

### 2.9. Metabolomics

Serum was collected from patients while they were fasting and stored at −80 C until being sent to Metabolon Inc (Morrisville, NC, USA) for processing as a single batch. Serum samples were processed and analyzed using an integrated analytic platform that consists of automated sample preparation, Liquid Chromatography/Gas Chromatography/Mass Spectrometry (LC/GC/MS), peak identification and deconvolution, and chemical intelligence. Data are curated by mass spectrometry analysts using specialized software. Biochemicals are identified by comparison to library entries of purified standards. Chromatographic properties and mass spectra allow matching to the specific compound or an isobaric entity using proprietary visualization and interpretation software. Peaks are quantified using area under the curve. Library matches for each compound are verified for each sample. Based on prior published works on serum metabolites and bariatric surgery, we focused our analysis to short chain fatty acids, branched chain amino acids, para-Cresol, phenylacetylglutamine, phenol, indoles, tryptophan-related metabolites, N-acetyl-putrescine, glutamate, and phosphatidylcholine [44,45,46,47].

### 2.10. Statistical Analysis

Clinical continuous variables were associated to each other using linear regression model in R. Means were compared using a univariate analysis of variance in R. Normality of data was tested using the Shapiro–Wilk method by using the function shapiro.test in R. Homogeneity of variance was tested using the F-test by using the function var.test in R. Categorical data were compared using the Fisher’s exact test in R. All means are expressed along with their standard deviations.

Multilevel sparse partial least square linear discriminant analysis (sPLS-DA) was done to analyze microbiome data using the Mixomics package in R (http://www.R-project.org). sPLS-DA identifies amplicon sequence variants that discriminates subjects by categories by simultaneously performing feature selection and modeling using lasso penalization. sPLS-DA operates using a supervised framework to find linear combinations of a limited set of variables, here amplicon sequence variants, that predicts altered resting state pairwise signaling, similar to prior published works [48]. In summary, microbial data were transformed into relative abundances and analyzed similar to the pathway described on www.mixomics.org (case study: Koren diverse bodysites). Two components were retained after error analysis. Forty amplicon sequence variants were retained in the model. Performance of the model was evaluated using the *perf* function in Mixomics which employs a 5-fold cross-validation with 100 repeats. Accuracy of the model was assessed using *auroc* function of Mixomics which graphs the area under the receiver operating curve of the model. Alpha diversity was measured using the Shannon Index, which measures species evenness. Data were rarefied for alpha diversity measurements to a depth of 60,709 sequences. Alpha diversity was tested using analysis of variance in R (formula: shannon~brain connection of interest). Differential abundance testing of microbiome data was performed using DESEq2 in R, which employs a Bayesian approach to fit non-rarified count data to a negative binomial model [49]. *p*-values were converted to *q*-values to correct for multiple hypothesis testing and a *q*-value < 0.05 was deemed significant [50]. 

Brain voxel morphometry at 6 months compared to baseline was analyzed using a general linear model (GLM) with permutation-based testing at 5000 iterations to create clusters with the Threshold Free Cluster Enhancement (TFCE) method [51]. All significance testing was conducted at *p* < 0.05, corrected for multiple comparisons using the false discovery rate (FDR) method. Images of results were produced in Mango software (http://ric.uthscsa.edu/mango). Resting state pairwise connections at 6 months compared to baseline was analyzed using univariate analysis of variance adjusting for multiple hypothesis testing using the FDR method. Similar to above, normality of data was tested using the Shapiro–Wilk method by using the function shapiro.test in R. The homogeneity of variance was tested using the F-test by using the function var.test in R.

Correlations between metabolites and brain connectivity was assessed using linear regression adjusting for multiple hypothesis testing by using the FDR method. All statistical analysis was done using R.

## 3. Results

### 3.1. LSG Reduces Measures of Obesity and Maladaptive Eating Behavior

**Figure 1:**
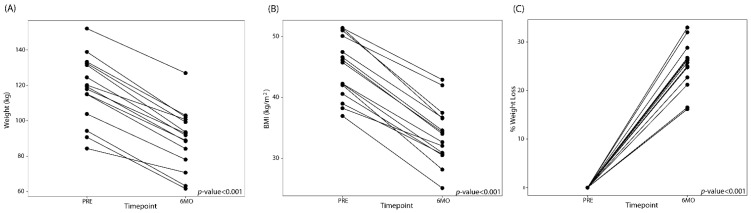
Line plots showing the (**A**) weight change, (**B**) changes in BMI, and (**C**) total percent body weight loss after laparoscopic sleeve gastrectomy (LSG). PRE: Before LSG, 6M0: 6 months post-LSG.

Eighteen subjects were enrolled in the study, of which, 14 underwent brain imaging. The average age of the participants were 37.4 years old + 9.7 (Table 1). The average weight and BMI before surgery was 119.4 + 19.8kg and 45.5 kg/m^2^ + 4.9, respectively. The demographic makeup of the participants is described in Table 1. LSG led to significant reductions in weight at 6 months (Figure 1). The average percentage of total body weight loss was 24.3% + 5.2 at 6 months after surgery.

There was a significant reduction in maladaptive eating behaviors as measured by YFAS symptom count (*p*-value < 0.001) and TFEQ_R21 as well as a reduction in anxiety and depression scores post bariatric surgery (Table 2). Three subjects reached criteria for food addiction diagnosis pre-surgery, but no patients fulfilled the criteria for food addiction after surgery (*p* = 0.22).

### 3.2. LSG Induces Changes in Morphology and Brain Connectivity that Are Associated with Eating Behavior

By analyzing the functional and structural brain MRI of patients before and after surgery, we saw significant changes in important reward regions including the amygdala and the putamen.

**Figure 2:**
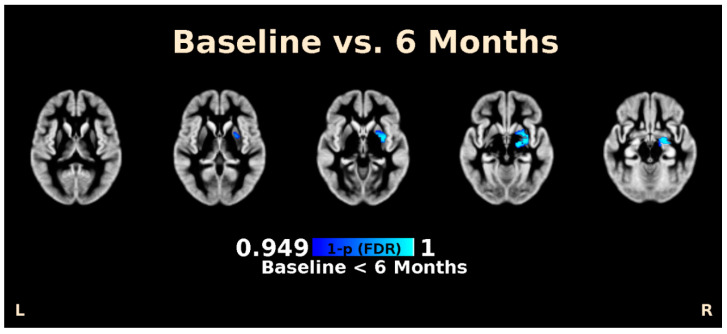
Voxel-based morphometry (VBM) analysis of brain MRI imaging of patients at baseline and 6 months post laparoscopic sleeve gastrectomy. Images above shows significant increases in brain volume in the amygdala and putamen after adjusting for false discovery rate (FDR). Inferior to superior cross-sectional images are presented from left to right.

Voxel-based morphometry analysis showed that subjects displayed a significantly higher gray matter volume in a cluster consisting of the putamen and amygdala after 6 months compared to baseline (*t* = 5.30, *p*_(*FDR*)_ = 0.002, cluster size = 518 voxels, MNI: X = 18, Y = −10, Z = −8) (Figure 2). Changes in voxel-based morphometry was not associated with any changes in eating behaviors, anxiety, or depression.

**Figure 3:**
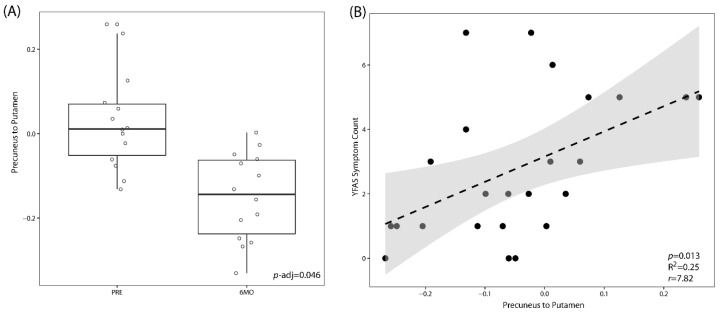
LSG leads to a significant decrease in the resting-state connectivity between the precuneus and the putamen. (**A**) Box plot showing resting-state connectivity between the precuneus and the putamen at baseline (PRE) versus those at 6 months (6MO) post bariatric surgery. *p*-Values are adjusted for multiple hypothesis testing. (**B**) Linear correlation between resting state connectivity between the precuneus and the putamen to food addiction behavior as measured by the Yale Food Addiction Scale (YFAS).

By analyzing the resting state pairwise connectivity of the brain, we found that patients had lower connectivity between the precuneus and the putamen at 6 months after surgery compared to baseline (*p*.adj = 0.046). The connectivity between the precuneus to the putamen across both timepoints was positively correlated to the YFAS symptom count (*p*-Value = 0.013) (Figure 3). This change in brain connectivity was not significantly correlated with either scores for anxiety or depression or any of the subscales of the TFEQ-R21.

### 3.3. LSG Induced Changes at the Reward Network Are Associated with Shifts in the Gut Microbiome and Circulating Metabolites

**Figure 4:**
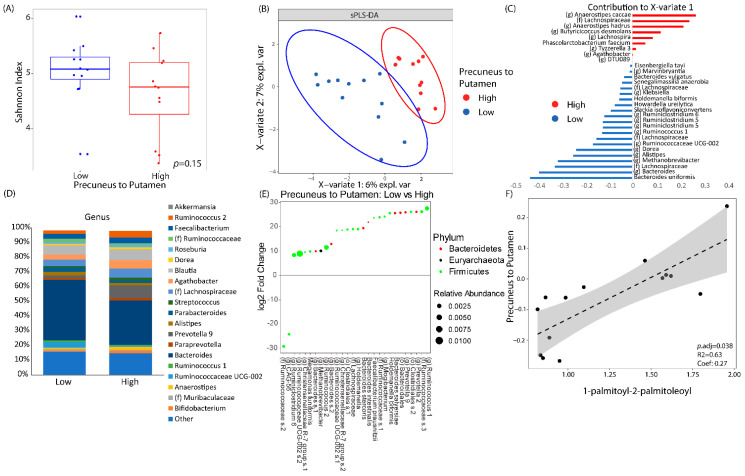
Brain connectivity was associated with significant changes in the fecal microbiome and serum metabolomics. Resting-state connectivity between the precuneus and the putamen were dichotomized as either high vs low based on its median value. (**A**) Alpha diversity between low vs high brain connectivity as measured by Shannon Index (a metric of species evenness). (**B**) Sparse partial least square discriminant analysis (sPLS-DA) plot showing how the gut microbiome can discriminate between patients with low or high connectivity. (**C**) The amplicon sequence variants that contributed to X-variate 1 of the sPLS-DA plot. (**D**) Taxonomic plots by genus of microbial communities between patients with low vs high connectivity between the precuneus and the putamen. Genera are only listed if they had a relative abundance of at least 1%. (**E**) Differential abundance testing as performed by DESEq2 adjusting for time showing log2 fold change in microbes of patients with low connectivity versus those with high connectivity. (**F**) Linear correlation between resting state connectivity between the precuneus and the putamen to 1-palmitoyl-2-palmitoleoyl, a phosphatidylcholine metabolite.

We then examined whether the brain connectivity between the putamen and precuneus was associated with any microbial or serum metabolite changes induced by surgery. By using the median value, resting state connectivity between the precuneus and the putamen were categorized as either high or low connectivity. There was no significant difference between subjects with a low versus a high connectivity in regard to alpha diversity. However, subjects with a higher connectivity between the precuneus and putamen did have a trend towards a lower alpha diversity than subjects with a lower level of connectivity (Figure 4A). Through the supervised learning model, sPLS-DA, subjects with low connectivity separated from subjects with high connectivity (Figure 4B) with an area under the receiver operating curve of 0.97. Thirty separate amplicon sequence variants contributed to the variation seen in the first component axis (Figure 4C). Of these 30 amplicon sequence variants, Bacteroides and Lachnospiraceae were associated the most to a low connectivity, while Anaerostipes was associated the most with a high connectivity. The taxonomic profiles are summarized in Figure 4D between patients with low or high connectivity. Differential abundance testing adjusting for time showed 28 different taxa associated with the connectivity between the precuneus and the putamen. An undefined species belonging to the family Ruminococcaceae and genus CAG-56 were associated with high connectivity while the other 26 taxa were associated with a low connectivity. Notable taxa belonging to those 26 associated with a low connectivity included 5 Bacteroides species, 2 Ruminococcus species, and Holdemanella, a microbe that was also associated with low connectivity, as determined by sPLS-DA (Figure 4E). Only one serum metabolite was significantly correlated with the connectivity between the precuneus and the putamen after adjusting for multiple hypothesis testing: 1-palmitoyl-2-palmitoleoyl, a phosphatidylcholine-related metabolite, was positively associated with the connectivity between the precuneus and the putamen (*p*.adj = 0.038, *r* = 0.63). There was no relationship between 1-palmitoyl-2-palmitoleoyl with any patient questionnaire data.

### 3.4. Associations Between Eating Behaviors with Shifts in the Gut Microbiome and Circulating Metabolites

**Figure 5:**
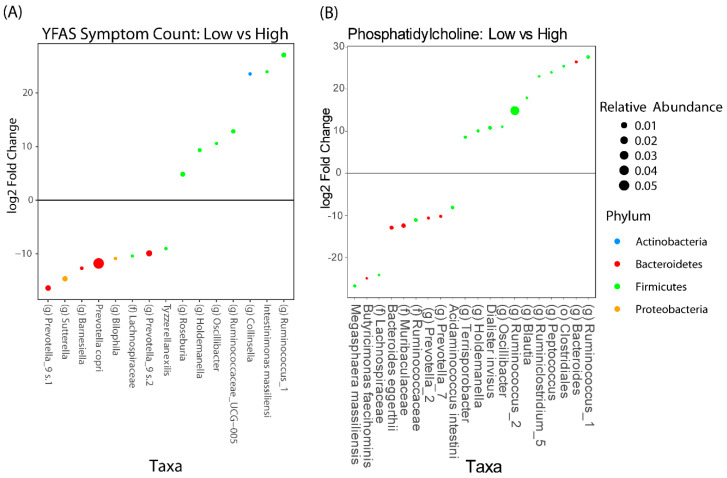
DESEq2 analysis adjusted for timepoints, showing the taxa that are associated with (**A**) YFAS symptom count and (**B**) phosphatidylcholine metabolite and 1-palmitoyl-2-palmitoleoyl. Taxa that have a positive fold change are those that are associated with a lower YFAS symptom count or 1-palmitoyl-2-palmitoleoyl level, respectively.

We then performed differential abundance testing with DESEq2 to see which microbes were associated with YFAS symptom count and levels of 1-palmitoyl-2-palmitoleoyl. Similar to above, we dichotomized the YFAS symptom count as either high or low by using a cutoff of three and we dichotomized the levels of 1-palmitoyl-2-palmitoleoyl as high or low by using the median value. The microbes that were associated with YFAS and 1-palmitoyl-2-palmitoleoyl are summarized in Figure 5. There were eight taxa that were positively associated with YFAS and seven taxa that were negatively associated. The two most abundant taxa belonged to the genus *Prevotella* and were both positively associated with YFAS. The most abundant taxa that was negatively associated belonged to the family Ruminococcaceae. Nine taxa were positively associated with 1-palmitoyl-2-palmitoleoyl and 11 taxa were negatively associated. Of the 20 taxa, the one with the greatest relative abundance was one belonging to the genus *Ruminococcus* and it was negatively associated with 1-palmitoyl-2-palmitoleoyl.

**Figure 6:**
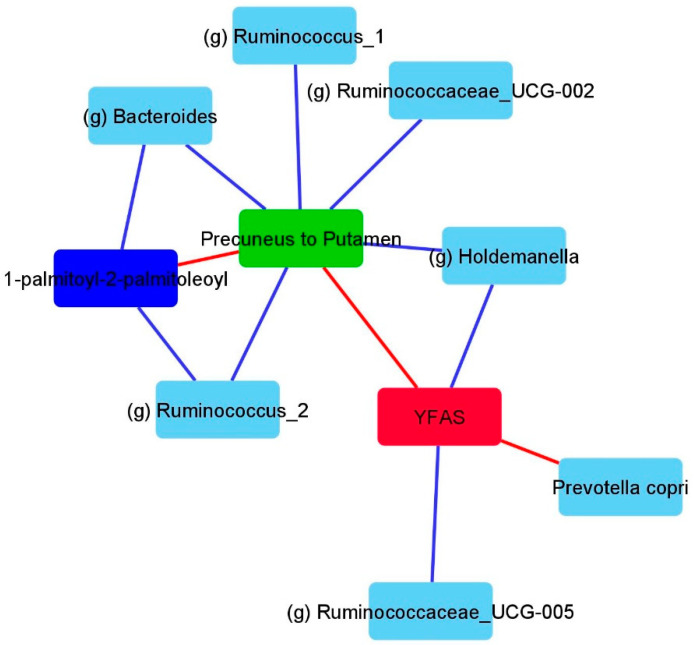
Summary figure depicting a subset of the associations across brain imaging, microbiome, metabolite, and eating behavior. This figure was solely created based on analysis, as previously mentioned above. Blue lines depict negative associations and red lines depict positive associations. YFAS: Yale Food Addiction Score.

A summary figure of the previously mentioned analysis representing all of the key associations between the brain, microbes, metabolites, and eating behavior is shown in Figure 6.

## 4. Discussion

This is one of the first studies to examine the interconnections between bariatric surgery and eating behavior through the brain–gut–microbiome axis. Despite the small sample size, this study showed a significant association between improvement in eating behaviors induced by bariatric surgery with changes in brain function at the putamen, a core reward region, and with shifts in gut microbiome composition.

In our study, we show that LSG decreased anxiety and depression scores as well as improved eating behavior as measured by the Yale Food Addiction Scale and the Three-Factor Eating Questionnaire. There is ample evidence that easy access to energy-dense and highly palatable foods play a major factor in the development of the current obesity epidemic [52]. Hedonic overeating is defined as eating beyond metabolic requirements from the expectation of or from the actual pleasure derived from consuming highly palatable foods. In obesity, the neuronal mechanisms that modulate the motivation to consume food are disrupted, resulting in impaired control and overconsumption of highly hedonic foods [53]. In prior published studies, bariatric surgery often leads to changes in food preference, meal frequency and meal size [54,55]. The exact reason for this change is likely multifactorial and includes alterations in appetite regulating gastrointestinal hormones, patient motivation, and changes in central nervous signaling [54,56]. Similar to other studies, we found, among our study subjects, a high incidence of addiction-like behaviors to food which were improved after surgery [57]. We also found that the proportion of subjects that fulfill the criteria for food addiction as defined by the YFAS decreased from 16.7% to 0% after surgery. Although the term food addiction is controversial, it is true that in obesity there is a disruption in the function of the brain’s reward network which is characterized by heightened response to food cues in core reward regions, a finding that is very similar to those seen in drug addiction [53,58]. Interestingly, bariatric surgery, mainly RYGB and LSG, are able to modify obesity-related alterations in brain function within the reward centers [20,56].

In line with these findings, we saw changes in both brain morphometric and connectivity within the reward network in our subjects after surgery. LSG led to an increase in amygdala and putamen volumes. Moreover, in this study, LSG induced a decrease in the connectivity between the precuneus and the putamen, which was linearly related to eating behaviors, as measured by the YFAS score. The putamen is located at the dorsal striatum, which is part of the extended reward network of the brain. In imaging studies, the putamen and the amygdala display higher reactivity to high-calorie food cues among obese subjects than among lean subjects [59,60]. The putamen is also preferentially activated during the consumption of drinks with a high sugar content [61]. The precuneus is involved in a variety of functions which include perception, cue reactivity, and salience. Studies in addiction have shown that connections to the precuneus were positively associated with nicotine dependence [62]. Our group found that, in healthy volunteers, anatomical connectivity between the putamen and the precuneus were key regions contributing to the discrimination in overweight/obese individuals from normal weight individuals [33]. Altered connectivity between the putamen and the precuneus has also been observed in obese women with food addiction behaviors [63]. Therefore, the reduction in connectivity seen in the present study between the precuneus and the putamen post bariatric surgery is in line with previous published data and suggests a possible central mechanism behind bariatric surgery induced changes in eating behavior, leading to weight loss.

In previous clinical trials, anxiety and depression metrics would often decrease after bariatric surgery [64,65]. The cause for this improvement is often attributed to a better self-image, an improvement in self-reported physical health, and a subjective increase in more positive social interactions [65]. A large body of evidence indicates that certain negative emotions such as depression, loneliness, and anxiety, leads to a tendency to overeat in obese individuals compared to normal-weight individuals [66]. To what extent changes in mood improve eating behaviors after bariatric surgery or whether surgically induced weigh loss improves mood cannot be answered in this study. There is, however, a body of evidence linking the gut microbiome to anxiety-related behaviors [67]. Thus the possible effect of bariatric surgery on the gut microbiome and its effect on anxiety/depression is a field that requires further exploration.

Because there is a direct effect of bariatric surgery on the gut microbiome, we wanted to further explore the connection between this altered morphometry and connectivity in the brain to changes in the gut microbiome. This study showed that the reduced connectivity between the precuneus and the putamen was associated with significant changes in the gut microbiome. By using sPLS-DA, we found that the gut microbiome can be used to discriminate among subjects with low or high connectivity between the precuneus and the putamen. The microbiome that distinguished those patients with low connectivity comprised of several species belonging to *Bacteroides*, *Ruminococcus*, and *Holdemanella*. The sPLS-DA model was further confirmed when we did an unbiased differential abundance testing with DESEq2. With a DESEq2 analysis adjusting for time, we showed that the associations between *Bacteroides*, *Ruminococcus*, and *Holdemanella* and the brain connectivity were independent of the effect of bariatric surgery. Similarly, we found associations between the gut microbiome, specifically *Holdemanella* and *Ruminococcus*, and YFAS symptom score. Human and mouse studies have shown that *Bacteroides* is associated with a lower BMI and leaner weight [68]. Prior studies of bariatric surgery patients have shown that those with the most significant weight loss were those that had higher levels of *Bacteroides* in stools [18]. Similarly, in a prospective trial, *Bacteroides richness* was higher in those individuals that were able to achieve sustained weight loss compared to overweight individuals [69]. Similarly, *Ruminococcus* has been associated with a lean body mass and a diet that is higher in fiber [70]. In another study of 98 participants, researchers found a significant reduction in *Ruminococcus* in patients who were obese or overweight compared to healthy controls [71]. While the data on *Holdemanella* is not as robust as *Ruminococcus* and *Bacteroides*, one study examining the role of avocado in weight loss did see a rise in *Holdemanella* with weight loss and avocado supplementation [72]. Therefore, the microbial associates we ascribed to food addiction and the connectivity of the precuneus and putamen are in line with the available data that associate the gut microbiome to obesity.

In addition to microbiome changes, bariatric surgery is also known to produce significant changes in serum metabolites. In our data, we see that a phosphatidylcholine metabolite, 1-palmitoyl-2-palmitoleoyl, was positively associated with the connection between the precuneus and the putamen. We also show that 1-palmitoyl-2-palmitoleoyl was also negatively associated with microbes belonging to the genus *Bacteroides* and *Ruminoccocus*, the same microbes that were negatively associated with the brain connections between the precuneus and the putamen. Phosphatidylcholines are major components in cell membrane and are involved in a variety of pathways including lipid metabolism and insulin sensitivity [73]. In several studies of bariatric surgery patients, the levels of phosphatidylcholine significantly decrease alongside weight loss and the level of phosphatidylcholine was positively associated with increased cholesterol transportation [44,74]. In addition to being related to obesity, studies have also shown phosphatidylcholine to be an integral compound in such neurological processes as dementia and acetylcholine synthesis [75,76]. Therefore, by showing a strong relationship between phosphatidylcholine and the connections related to the putamen, our data, alongside prior published works, suggests that decreases in phosphatidylcholine may play a critical role in the changes in the brain function seen after surgery, and these changes may be potentially linked to the gut microbiome, although the mechanism behind the latter link is not clear. Gut microbiome participates in the metabolism of dietary phosphatidylcholine, and its byproducts have been linked to atherosclerosis. However, to what extent the decrease in this metabolite in the host serum is due to changes in diet and/or gut microbiome–host metabolic interactions needs to be further explored.

We would like to acknowledge four important limitations of the current study. First, the main limitation to this study is its small sample size. Despite this, we were able to show significant changes relating bariatric surgery to the brain–gut–microbiome axis. Moreover, the longitudinal design of the study partially compensates for this shortcoming and gives a sense of the directionality of the associations. Second, we assessed eating behaviors and more specifically addictive-like eating behavior with the YFAS symptom score, which has a limited range (0–7) and may not be as sensitive to detect changes in other eating behaviors including impulsive eating behaviors. In addition, because of the small sample size, we could not control for the effect of changes in mood (anxiety, depression) or in appetite/gut peptide levels on the YFAS score after surgery. Third, because of the design and small sample size, we were not able to assess for the effect of dietary changes on the gut microbiome and metabolites. Furthermore, sPLS-DA modeling of the microbiome does tend to overfit data with small sample sizes. We have tried to overcome this limitation by also performing unbiased differential abundance testing with DESEq2 to confirm the findings from the sPLS-DA model. However, future larger studies should be performed to validate the findings found in our model. Fourth, our population comprises a relatively small number of women aged 18 to 55, without major comorbidities, and therefore our findings cannot be extrapolated to other obese populations. The strengths of our study include a well-characterized population, followed closely for 6 months. Data and samples were prospectively collected in a standard manner. This data included eating behavior questionnaires, anthropometrics, high-resolution fMRI of the brain, stool samples for high-depth sequencing using up to date analysis techniques that provide better taxonomic resolution, and well-characterized serum metabolite data.

## 5. Conclusions

In conclusion, this is one of the few studies to date to examine the effects of bariatric surgery on eating behavior using a multi-omics approach. We believe that our study builds on prior published works to show how bariatric surgery can affect eating behavior through alterations in the gut microbiome, serum metabolites, and brain morphometry and connectivity. While these results may shed new light on novel pathways to aid patients with weight loss, they should be validated in a larger cohort of patients that includes both men and women. Future studies will also be required to further investigate the mechanisms behind these BGM interactions following bariatric surgery.

## Figures and Tables

**Table 1:** Baseline patient characteristics.

Average (SD) (*n* = 18)	Pre-Surgery
Age (y)	37.4 (9.7)
BMI (kg/m^2^)	45.5 (4.9)
Weight (kg)	119.4 (19.8)
Race/Ethnicity	
Non-Hispanic White (%)	44.4
African American (%)	11.1
Asian (%)	11.1
Hispanic (%)	33.3

SD: standard deviation; y: year; BMI: Body Mass Index.

**Table 2:** Patient questionnaire results.

Mean (Standard Deviation)	Pre-Surgery	Post-Surgery (6 mo)	*p*-Value
YFAS Symptom Count	3.7 (2.1)	1.6 (1.0)	<0.001
Food Addiction (No. of Patients)	3	0	0.22
HADS Anxiety	7.2 (3.2)	5.3 (3.9)	0.01
HADS Depression	4.5 (3.3)	1.9 (3.3)	0.008
TFEQ CR	3.0 (0.3)	3.1 (0.5)	0.07
TFEQ UE	2.3 (0.5)	1.7 (0.5)	0.02
TFEQ EE	2.1 (1.0)	1.7 (0.7)	0.06

YFAS: Yale Food Addiction Scale; HADS: Hospital Anxiety and Depression Scale, TFEQ: Three-Factor Eating Questionnaire’ CR: cognitive restraint; UE: uncontrolled eating; EE: emotional eating. 6 mo: 6 months post-surgery.
